# Association of the TNFRSF1A genotype with lung function impairment in patients with sarcoidosis

**DOI:** 10.1038/s41598-026-51077-x

**Published:** 2026-05-05

**Authors:** A. G. Lunde, S. Romundstad, A. H. Henriksen, H. Sorger, E. Boerner, N. Schröder, F. Bonella

**Affiliations:** 1 Department of Clinical and Molecular Medicine, Faculty of Medicine and Health Sciences, Norwegian University of Science and Technology (NTNU), Trondheim, Norway; 2 Levanger Hospital, Nord-Trondelag Hospital Trust - Levanger, Levanger, Norway; 3 Clinic of Medicine, St. Olav’s Hospital, Trondheim University Hospital, Trondheim, Norway; 4 Department of Circulation and Medical Imaging, Faculty of Medicine and Health Sciences, Norwegian University of Science and Technology (NTNU), Trondheim, Norway; 5 Center for Interstitial and Rare Lung Diseases, Pulmonology Department, Ruhrlandklinik University Hospital, University of Duisburg-Essen, Essen, Germany; 6 Department of Medicine, University Hospital of North-Norway, Harstad, St.Olavs gate 70, Harstad, 9406 Norway

**Keywords:** Sarcoidosis, TNF-α, TNFR1, rs1800693, Lung function, Prognosis, Biomarkers, Diseases, Genetics, Medical research

## Abstract

The single-nucleotide polymorphism (SNP) rs1800693 in the tumor necrosis factor receptor 1 (TNFR1) gene leads to the formation of a protein-mimicking effect of TNF-α inhibitors, which are agents used to treat sarcoidosis. We investigated the association between rs1800693 and disease progression in two populations with non-Lofgren sarcoidosis (nLS). We genotyped two nLS cohorts (Ruhrlandklinik, Germany, *n* = 108, and the HUNT study, Norway, *n* = 393), and two control groups with (*n* = 313) and without (*n* = 2414) COPD for rs1800693 (A/G polymorphism). Pulmonary function tests at inclusion and at the end of follow-up were compared according to genotype. The mean follow-up times were 6.5 (German) and 7.9 (Norwegian) years. German AA patients had an absolute decline in %pred FVC (-8.5, 95% CI -3.4, -13.6) and DLco (-4.9, 95% CI -0.1, -9.7). In AG/GG (G+), the absolute decline in FVC was less pronounced (-3.9, 95% CI -1.1, -6.8), and DLco was stable. In the Norwegian patients, the decrease in lung function was similar between the AA and G+ groups. Compared with homozygous GG, homozygous AA was associated with a lower FVC %pred at baseline (-5.7, 95% CI -0.2, -11.6) and at the end of follow-up (-7.0, 95% CI -0.1, -14.0). Genotype was not associated with lung function in either control group. TNFR1 rs1800693 might be associated with lung function impairment and decline in non-Lofgren sarcoidosis patients.

**Supplementary Information:**

The online version contains supplementary material available at 10.1038/s41598-026-51077-x.

## Introduction

Sarcoidosis is an immune-mediated, systemic disorder characterized by granulomatous inflammation, most commonly in the lungs and intrathoracic lymph nodes^1^. One of the two main phenotypes of sarcoidosis, Lofgren syndrome (LS), is characterized by the classic triad of fever, arthritis and hilar adenopathy and is associated with spontaneous remission^2^. The other phenotype, non-Lofgren syndrome (nLS), has a variable presentation where the disease burden and prognosis are related to organ involvement and damage^3^. Pulmonary sarcoidosis, which affects more than 90% of patients with nLS, can lead to deterioration of pulmonary function, irreversible fibrosis and increased risk of death^4,5^. The mechanism by which granulomatous inflammation causes lung damage is driven by a complex immunological process in which tumor necrosis factor alpha (TNF-α) plays a major role^6^. TNF-α inhibitors, such as infliximab, can improve pulmonary function and are used to treat severe pulmonary sarcoidosis^7–9^.

Genome-wide association studies (GWASs) have identified genes associated with both susceptibility and prognosis in patients with sarcoidosis^10–12^. However, to improve our understanding of its pathogenesis, the corresponding biological relevance of an identified gene needs to be determined. The activation of immune cells involved in sarcoidosis inflammation, such as lymphocytes and macrophages, is mediated by TNF-α through the binding of the cytokine to one of its main receptors, TNFR1. One of the polymorphisms of the TNFR1 gene, rs1800693, has been associated with susceptibility to multiple sclerosis^13^. An experimental study of the functional outcome of the alternative allele (G) revealed the skipping of exon 6 and premature termination of transcription in approximately 10% of messenger RNAs (mRNAs). This polymorphism generates an altered and soluble isoform, named Δ-6 TNFR1, with the capacity to neutralize TNF-α^14^. Increasing levels of mRNA expression of the Δ-6 TNFR1 isoform correlate with copies of the alternative allele (G), possibly inhibiting TNF-α in a dose‒dependent manner^15^. The polymorphism of the TNFR1 gene has been associated with lower CRP levels in summary statistics GWASs and has been used as an instrumental variable in Mendelian randomization studies of other chronic diseases^16^.

Linkage of genetic variants to disease traits could compensate for the shortage of prognostic markers in sarcoidosis. The discovery of the human leukocyte antigen (HLA)-DRB1*0301 and its association with a favourable prognosis has led to HLA phenotyping to guide treatment decisions in certain settings^10^. Other genetic markers with established downstream molecular and cellular processes have the potential to serve as early prognostic markers, provide grounds for clinical trials and guide treatment decisions^17^.

Our study aimed to investigate the genotype distributions of the common (A) and alternative (G) alleles of the single-nucleotide polymorphism (SNP) rs1800693 and their associations with disease trajectories in two different cohorts of nLS patients from Germany and Norway. We included two control groups with no suspected effect on lung function by TNF-α activity, one with chronic obstructive pulmonary disease (COPD) and one with no lung disease^18^.

## Results

### Baseline characteristics

The allele frequency was similar among German nLS, Norwegian nLS and the two control groups (*p* = 0.35) (Table 1). Compared with the Norwegian nLS group, the German nLS group was younger (mean difference 9.3 years, 95% CI 6.3, 12.4) and more often on immunosuppressant treatment (*p* < 0.001). The distribution of Scadding stages differed, with more stage I patients in the Norwegian group (*p* < 0.001). FVC and DLco were lower in the German group, with mean differences in % pred of 5.9 (95% CI 1.8, 10.1) and 14.2 (95% CI 9.8, 18.6), respectively. We found no difference in Scadding stage, extrapulmonary disease or treatment status according to genotype in either of the sarcoidosis populations. We could not make a reliable estimate of differences in smoking exposure due to many missing values in the German nLS group.

**Table 1:** Baseline characteristics of the study population.

	Non Lofgren sarcoidosis Essen *n* = 108	Non Lofgren sarcoidosis HUNT *n* = 393	Control group 1*, HUNT *n* = 313	Control group 2†, HUNT *n* = 2414
Gender, male/female	66/42 (61/39)	248/145 (63/37)	157/156 (50/50)	1045/1369 (43/57)
Age, years	42.6 (± 11.8)	51.9 (± 14.5)	47.2 (± 13.9)	46.2 (± 12.9)
Genotype, rs1800693				
AA	34 (32)	164 (42)	137 (44)	1019 (42)
AG	54 (50)	179 (46)	141 (45)	1063 (44)
GG	20 (18)	50 (13)	35 (11)	332 (14)
BMI	28.7 (± 5.6)	27.3 (± 4.6)	25.9 (± 3.7)	26.7 (± 4.1)
*missing*	*11 (11.0)*	*0*	*1 (0.0)*	*3 (0.0)*
Pulmonary function tests				
FVC, % predicted	87.7 (± 18.3)	93.6 (± 16.8)	93.3 (± 16.6)	100.9 (± 11.6)
*missing*	*27 (25.0)*	*25 (6)*	*0*	*0*
DLco, % predicted	72.1 (± 14.0)	86.3 (± 21.0)		
*missing*	*11 (9.3)*	*34 (8.7)*		
Scadding stage				
0	3 (3)	34 (8)		
I	21 (19)	125 (32)		
II	59 (55)	109 (28)		
III	17 (16)	80 (20)		
IV	8 (7)	45 (12)		
Organ involvement				
Skin	21 (19)	60 (15)		
Liver	7 (7)	13 (3)		
Heart	11 (10)	14 (4)		
Extrapulmonary disease	57 (49)	165 (42)		
Treatment, yes/no	93/15 (86/14)	215/178 (55/45)		
Corticosteroids	51 (47)	210 (53)	54 (18)	137 (5)
Combination with DMARDs	42 (39)	39 (10)		
ACE, U/L	42 (43)	80 (66)		
*missing*	0	*10 (5)*		
Smoking				
Never	15 (14)	239 (61)	79 (25)	1027 (43)
Former	25 (23)	110 (28)	64 (20)	629 (26)
Current	7 (7)	44 (11)	170 (54)	757 (31)
*missing*	*61 (56)*	*0*	*0*	*1 (0.0)*

Data are collected from health records for nLS (Essen and HUNT) and from HUNT for control groups 1 and 2. Data are presented as the mean ± SD, median (IQR) or n (%). No missing values unless otherwise stated. HUNT: The Trøndelag Health Study. BMI: Body mass index. FVC: Forced vital capacity. DLco Diffusing capacity of the lungs for carbon monoxide. DMARDs: Disease-modifying antirheumatic drugs, such as Azatioprin and Methotrexate. ACE: Angiotensin-converting enzyme. CRP: C-reactive protein. *: Control group with chronic obstructive pulmonary disease, from HUNT. †: Control group without lung disease, from HUNT.

**Fig. 1:**
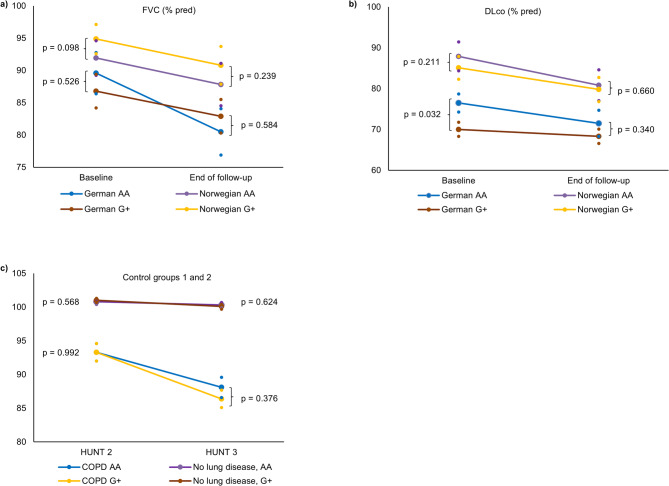
Mean plot of (**a**) FVC (% pred), and (**b**) DLco (% pred) in German and Norwegian nLS patients. Mean plot of (c) FVC (% pred) in control groups 1 and 2.

In the German nLS patients, the FVC % pred was similar for AA and G+ (-2.8, 95% CI -11.5, 5.9) at inclusion (Table 2). The DLco % pred was significantly lower in G+ than in AA (-6.5, 95% CI -0.6, -12.4). In the Norwegian nLS, the FVC % pred was not significantly greater in G+ than in AA (2.9, 95% CI -0.5, 6.4) (Fig. 1). Compared with homozygous AA, homozygous GG had a significantly greater FVC % pred (5.7, 95% CI 0.2, 11.6) but a similar DLco % pred (-2.8, 95% CI -7.2, 1.6) (Table 2).

**Table 2:** Lung function as % predicted at inclusion and end of follow-up, by genotype of TNFRSF1A. Mean difference (95% CI) between AA and G + and between AA and GG.

	Non Lofgren sarcoidosis *Essen n* = 108	Mean difference, 95% CI	Non Lofgren sarcoidosis HUNT *n* = 393	Mean difference, 95% CI
FVC, inclusion				
AA	89.6 (± 16.1)		91.9 (± 16.9)	
G+	86.8 (± 19.3)	-2.8 (-11.5, 5.9)	94.9 (± 16.8)	2.9 (-0.5, 6.4)
GG	79.9 (± 18.6)	-9.6 (-21.1, 1.8)	97.7 (± 17.5)	5.7 (0.2, 11.6)
*missing*	27		*25*	
FVC, end of follow-up				
AA	80.5 (± 19.2)		87.8 (± 20.4)	
G+	82.9 (± 18.3)	2.4 (-6.3, 11.1)	90.8 (± 21.3)	2.5 (-1.7, 6.7)
GG	84.3 (± 21.9)	3.7 (-9.3, 16.8)	94.9 (± 20.1)	7.0 (0.1, 14.0)
*missing*	*29*		*35*	
DLco, inclusion				
AA	76.5 (± 12.3)		87.9 (± 22.1)	
G+	70.0 (± 14.3)	-6.5 (-0.6, -12.4)	85.1 (± 20.2)	-2.8 (-7.2, 1.6)
GG	61.9 (± 13.5)	-14.6 (-7.0, -22.2)	85.1 (± 16.0)	-2.8 (-10.1, 4.4)
*missing*	*10*		*34*	
DLco, end of follow-up				
AA	71.5 (± 17.5)		80.8 (± 23.4)	
G+	68.3 (± 13.6)	-3.2 (-9.9, 3.4)	79.8 (± 21.2)	-1.1 (-5.8, 3.7)
GG	70.5 (± 13.9)	-1.0 (-11.2, 9.2)	81.0 (± 17.3)	0.2 (-7.6, 7.9)
*missing*	*18*		*45*	

Data are collected from health records and presented as the mean ± SD. No missing values unless otherwise stated. HUNT: The Trøndelag Health Study. FVC: Forced vital capacity. DLco: Diffusing capacity of the lungs for carbon monoxide.

In control group 1, the mean z score for FEV1 was − 1.95, and that for FEV1/FVC was − 2.44. The mean FEV1 as % pred was 72.6, which is compatible with stage II disease according to the Global Initiative for Chronic Obstructive Lung Disease (GOLD). A total of 79 (25%) were never smokers, of whom 65 (82.2%) reported exposure to passive smoking and the remaining reported chronic asthma or bronchitis on questionnaires. In control group 2, pulmonary function was within the normal range.

## Lung function changes by genotype

**Fig. 2:**
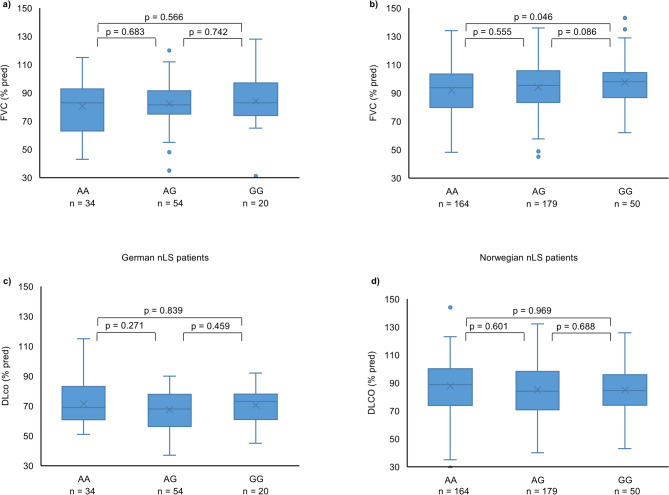
Box plots of FVC (% pred) at end of follow up according to genotypes in (**a**) German, and (**b**) Norwegian nLS patients. Box plots of DLco (% pred) at end of follow up according to genotypes in (**c**) German, and (**d**) Norwegian nLS patients.

The mean follow-up durations were 6.5 (± 4.9) and 7.9 (± 6.8) years in the German and Norwegian nLS groups, respectively. The mean follow-up for both control groups was 10.6 years (± 0.5). At the end of follow-up, no significant difference in FVC or DLco% pred was observed among genotypes AA and G + in either sarcoidosis population (Table 2; Fig. 1). However, in the Norwegian patients, the comparison of homozygous AA and GG genotypes revealed a significant difference in FVC % pred of 7.0 (95% CI 0.1, 14.0) (Table 2; Fig. 2).

Crude estimates of absolute and rate of decline in FVC and DLco % pred were analysed for participants with measurements at both inclusion and end of follow-up (Table 3 and Figure S1). In the German patients, homozygous AA had a significant absolute decline in FVC and DLco. In G+, the absolute decline in the FVC was less pronounced, although significant, while DLco was stable. In the homozygous GG genotype, the FVC was stable, and the DLco improved. Among the Norwegian patients, both the homozygous AA and G+ patients had significant absolute decreases in the FVC and DLco. In homozygous GG, FVC and DLco were stable.

**Table 3:** Crude estimates of absolute and rate of lung function decline as a percentage of the predicted value, by genotype of TNFRSF1A.

	FVC, absolute	FVC, annual rate	DLco, absolute	DLco, annual rate
nLS, Essen (*n* = 68)				
AA	-8.5 (-3.4, -13.6)	-1.9 (± 3.1)	-4.9 (-0.1, -9.7)	-0.8 (± 2.7)
G+	-3.9 (-1.1, -6.8)	0.0 (± 3.3)	-0.6 (-3.7, 2.5)	-0.5 (± 4.8)
GG	2.8 (-2.2, 7.7)	0.8 (± 3.2)	8.5 (2.3, 14.7)	2.1 (± 3.8)
nLS, HUNT (*n* = 365)				
AA	-3.5 (-1.1, -5.9)	-0.2 (± 4.5)	-7.0 (-3.8, -10.3)	0.8 (± 4.9)
G+	-4.5 (-2.5, -6.6)	0.2 (± 8.7)	-4.7 (-2.0, -7.4)	0.4 (± 5.9)
GG	-3.0 (-8.0, 2.0)	-0.3 (± 3.8)	-3.8 (-8.6, 1.0)	-0.1 (± 3.7)

Data are collected from health records. Lung function decline is marked by negative values. Data are presented as the mean difference with 95% confidence intervals and mean ± standard deviation. HUNT: The Trøndelag Health Study. FVC: Forced vital capacity. DLco: Diffusing capacity of the lungs for carbon monoxide.

In a linear mixed-effects model of FVC decline (% pred) adjusted for smoking and treatment status, we found no statistically significant difference between AA and G+ (0.09/year, 95% CI -0.18, 0.36) or between AA and GG (0.02/year, 95% CI -0.46, 0.41) in the Norwegian nLS population. In the German population, the decline in FVC % pred was greater for AA than for G+ (mean difference 1.21/year, 95% CI 0.08, 2.34) and GG (mean difference 1.73/year, 95% CI -0.66, -2.81). For DLco % pred, we found no statistically significant difference between AA and G + or between AA and GG in either population.

We found no difference in the FVC at inclusion or at the end of follow-up according to genotype in either control group (Table S1). The rate of decline in FVC % pred was similar in both the nLS groups and control group 1 (Figure S2 and S3).

## Discussion

In this study, we assessed the role of a polymorphism in the TNFR1 gene in lung function decline over 6–8 years in two different nLS populations. Compared with the alternative allele (G), the homozygous common allele (A) was associated with a lower FVC in the Norwegian patients and a more rapid decline in the German patients. We found no associations between genotype and other measures of severity, such as Scadding stage, extrapulmonary organ involvement, or need for treatment. The relevance of our findings was supported by a lack of association between genotype and lung function in people with COPD or without lung disease.

Several studies have demonstrated clear evidence for a genetic backdrop in sarcoidosis, but the genetic signatures might be specific for ancestry or a genetic‒environmental interplay in a unique population^19,20^. For rs1800693, a study from a tertiary centre for sarcoidosis in the Netherlands revealed that the AA genotype was associated with more severe disease, as measured by pulmonary function tests and biomarkers at baseline, and with a better treatment response to a TNF-α inhibitor^21^. This is in line with our findings in the German population. The lack of a significant association in a more diverse population of sarcoidosis patients might have several explanations.

Regardless of whether sarcoidosis is triggered by an exogenous antigen or a self-antigen, the heterogeneity in both the disease course and treatment response indicates that the response of the host is crucial. The many inflammatory pathways implicated in sarcoidosis may not be equally important in different patients or stages of the disease. TNF-α inhibition leads to improvement in severe sarcoidosis but might also trigger a sarcoid-like reaction in some patients with other autoimmune diseases treated with TNF-α inhibitors^22^. Greater TNF-α release from alveolar macrophages has been reported in sarcoidosis patients who are unresponsive to glucocorticoid treatment, as well as in patients with a progressive disease course, indicating that TNF-α contributes more strongly to inflammation in patients with progressive sarcoidosis than in those with milder variants^23,24^. The proportion of treated patients in the German nLS population was high, indicating more severe disease. This might explain the larger effect of the genotype on lung function decline compared to the Norwegian nLS population.

There was a significant difference in tobacco use and DLco levels between the two populations. Both experimental and observational studies have shown a possible attenuating effect of smoking on granulomatous inflammation, but smoking leads to other detrimental lung conditions, such as emphysema and fibrosis, both of which are evident by a decrease in DLco^25,26^. Smoking might also affect genetic polymorphisms. A gene‒environment interaction study in Sweden revealed that the association between smoking and the risk of sarcoidosis was modulated by the genetic signature, indicating that smokers and nonsmokers might have different risk profiles despite a shared genetic background^27^.

There is a paucity of prognostic markers to guide treatment decisions in patients with sarcoidosis. Despite substantial research efforts, no biomarker has shown adequate sensitivity or specificity to distinguish patients with or without benefits from treatment. Clinical risk factors for increased morbidity and mortality, such as pulmonary fibrosis, pulmonary hypertension and the involvement of vital organs, are signs of advanced disease with limited latitude to improve prognosis^28^. Specific HLA variants have been associated with disease behaviour, treatment response and toxicity^29^. Other SNPs from the TNF-α genetic domain have also been related to treatment response^21,30^. Linking clinical phenotypes and genetic markers might provide opportunities for the early identification of at-risk populations and tailored treatment.

The strengths of the study include a well-defined study population with detailed information on disease-specific and anthropometric measures and the opportunity to replicate the analyses to validate the results in four populations: two with nLS, one with COPD and one without lung disease. The HUNT study collected data from a genetically uniform population with similar environmental exposure. Additionally, the German and Norwegian nLS patients presented equal allele frequencies for rs1800693, supporting homogenic ancestry.

Our study also has limitations. Studies of nonparticipants in HUNT2 and HUNT3 patients revealed a lower socioeconomic status and more smokers and comorbidities^31^, which could mean that patients with more severe sarcoidosis might not have participated in the HUNT study. We suspect that some of the differences in DLco among the study populations were related to tobacco exposure. Given that all the study participants were of Caucasian heritage, our results might not be relevant to people of other ancestries. The association between rs1800693 and impaired lung function in nLS patients might be explained by the amelioration of TNF-α-driven inflammation, but other genes have also been associated with disease behaviour in patients with sarcoidosis^20^. Tests for linkage disequilibrium with other relevant polymorphisms, such as the HLA domain, were not within the scope of this study. Participants in both control groups had to be alive 10 years after inclusion to fulfil the criteria of a second spirometry, but we considered it less problematic, as survival was not the aim of this study.

We found an association between sarcoidosis and an SNP with well-established biological and functional effects on inflammation. The effect of this polymorphism is believed to be mediated through the inhibition of TNF-α, a cytokine implicated in more treatment-resistant and severe disease. Additional studies of disease trajectories according to the genotype rs1800693 could help clarify their relationship. Prospective studies of the treatment response to TNF-α inhibitors by genotype of rs1800693, as well as TNF G308-A, could guide therapeutic decision-making in severe sarcoidosis patients.

## Methods

### Study population

**Fig. 3:**
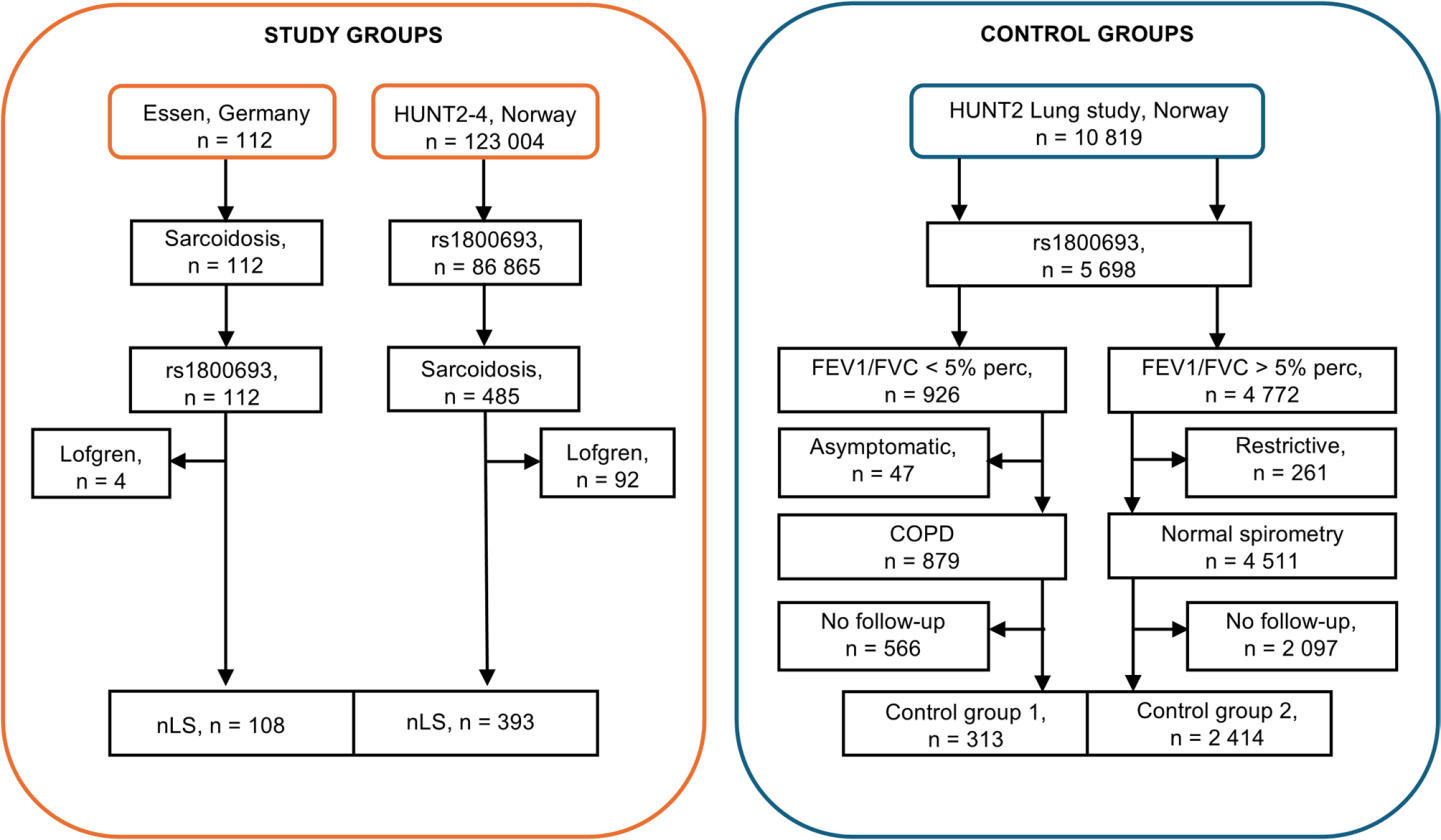
Flow chart. Inclusion and exclusion of study participants from the Ruhrlandklinik University Hospital and the Trøndelag Health Study (HUNT). LS: Lofgren sarcoidosis. nLS: Non-Lofgren sarcoidosis. FEV1: Forced expiratory volume in 1 s. FVC: Forced vital capacity. COPD: Chronic obstructive pulmonary disease.

We performed a retrospective case‒control study of two separate nLS populations from Essen, Germany, and the Trondelag Health Study (HUNT), Norway. Patients with nLS were included at the time of diagnosis and excluded at the end of follow-up (2026 for Norwegian nLS, 2015 for German nLS). We included two control groups in which we suspected that there was no association between lung function and TNF-α inhibition: one with COPD and a random sample without lung disease^18^. The control groups were included at the date of participation in the second HUNT (HUNT2; 1996–1998) and excluded at the date of participation in the third HUNT (HUNT3; 2006–2008) (Fig. 3).

The German study population included consecutive patients with sarcoidosis referred to the Centre for Interstitial and Rare Lunate Diseases, Ruhrlandklinik University Hospital, University of Duisburg-Essen, Essen, Germany. All the subjects provided written informed consent.

The Norwegian study population included patients with sarcoidosis who participated in the HUNT study. The HUNT study is a population-based health survey initiated in 1984, in which all inhabitants in the county aged 20 years or older have been invited to four surveys spanning more than three decades^32,33^. The surveys include clinical examinations and measurements, interviews and questionnaires, biochemical analyses of blood and urine, and biobanking. A complete GWAS was performed on approximately 88 000 participants from HUNT2-4^34,35^. In HUNT2 and HUNT3, spirometry was performed on a randomly selected sample (5% and 10%, respectively) and a sample reporting respiratory symptoms, medication or a diagnosis of obstructive lung disease.

Participants with sarcoidosis were identified by linkage with hospital records between 16.01.1984 and 06.01.2026, and health records were assessed to validate the diagnosis. From HUNT2-4, 393 participants with sarcoidosis were genotyped for rs1800693. Control group 1 included participants with COPD (FEV1/FVC z score < -1.64 and respiratory symptoms, *n* = 313). Control group 2 included participants with normal spirometry (*n* = 2414). Written informed consent was obtained from all HUNT participants.

The study was performed in accordance with the Helsinki declaration and was approved by the Regional Committee for Medical and Health Research Ethics (REK id 2019/655) in Norway and approved by the local Institutional Review Board in Essen, Germany (IRB nr: 21-9904-BO).

### Definition of non-Lofgren sarcoidosis

All study subjects with sarcoidosis were diagnosed according to the World Association for Sarcoidosis and Other Granulomatous Disorders (WASOG) criteria^1^. All sarcoidosis patients were classified as LS and nLS by clinical history and radiographic examinations, and according to Scadding stages^36^. Among the 112 sarcoidosis patients from Essen, 108 (96%) had nLS. From HUNT, 393 of 485 (81%) were nLS.

### Covariates

We collected information on sex, age, smoking status (former, current, never), body mass index (BMI) and comorbidities from health records. Pulmonary function test results were collected from health records for sarcoidosis patients and HUNT for controls. In HUNT2 and HUNT3, spirometry was performed according to the 1995 and 2005 American Thoracic Society guidelines and described in detail elsewhere^37–39^. Results are reported as percent of predicted (%pred) according to Global Lung Initiative 2012 (GLI 2012).

Sarcoidosis treatment regimes, results of histological and radiological investigations, as well as blood samples, were collected from hospital records.

### Genotyping

For HUNT participants, genotyping was performed at the Norwegian University of Science and Technology (NTNU) using the Illumina HumanCoreExome arrays (12 v.1.0, 12 v.1.1 and 24)^34,35^. In the German study population, genotyping was performed at the University Hospital Essen via the TaqMan SNP Genotyping Assay and the Applied Biosystems 7500 Fast RT‒PCR System (Life Technologies Corp. Carlsbad, California, USA). The SNP rs1800693 genotypes were in Hardy‒Weinberg equilibrium for both study populations.

### Statistical analyses

A chi-square table was used to compare the observed number of each genotype with the expected number for a population in Hardy‒Weinberg equilibrium (*p* > 0.05). We compared outcomes by the main exposure variable, genotypes AA and AG/GG (G+), as well as AA and GG. Categorical variables were analysed by the chi-square test. Changes in pulmonary function according to the FVC % pred and DLco % pred between inclusion and the end of follow-up within each separate group were analysed via paired t tests for participants with both measurements. Normality was tested by inspection of histograms and QQ-plots. Linear mixed-effects models with random intercepts were used to examine differences in lung function decline between genotypes AA/G + and AA/GG in the separate study populations. The observation time (years), genotype, and their interaction were included as fixed effects. Analyses were adjusted for smoking and treatment status (never/ever). The time‒by‒group interaction term was used to estimate the difference in annual decline between genotypes. The statistical analyses were performed via the Statistical Package for the Social Sciences (SPSS), version 30.0.

## Supplementary Information

Below is the link to the electronic supplementary material.

Supplementary Material 1

## Data Availability

The data used in this study were provided by the Ruhrlandklinik University Hospital, Essen and HUNT Research Centre. Data from Ruhrlandklinik are available upon request. Data from the HUNT Research Centre are available for researchers affiliated with institutions in Norway or abroad on application ( [https://www.ntnu.edu/hunt](https:/www.ntnu.edu/hunt) ).
