# Supplementary material for: Association of the TNFRSF1A genotype with lung function impairment in patients with sarcoidosis
Source: Sci Rep. 2026 May 5;16:20669. doi: 10.1038/s41598-026-51077-x (PMC13333820; doi:10.1038/s41598-026-51077-x)
Supplement: Supplementary file 1 — Supplementary Material 1 [file 41598_2026_51077_MOESM1_ESM.pdf]

SUPPORTING INFORMATION

Lung function in control Norwegian (HUNT) and German control groups

Table S1. Lung function (FVC % pred) at inclusion and end of follow-up in the two control groups, according to genotypes of TNFRSF1A

	**Control group 1*, HUNT**<br>n = 313	Mean difference, 95% CI	**Control group 2†, HUNT**<br>n = 2414	Mean difference, 95% CI
FVC, inclusion				
AA	93.3 (±16.6)		100.8 (±11.5)	
G+	93.3 (±17.3)	0.0 (-3.7, 3.7)	101.0 (±11.6)	0.3 (-0.7, 1.2)
GG	91.0(±16.2)	-2.3 (-8.2, 3.6)	101.0 (±11.3)	0.3 (-1.1, 1.7)
FVC, end of follow-up				
AA	88.1 (±17.2)		100.3 (±12.8)	
G+	86.4 (±17.3)	-1.7 (-5.6, 2.1)	100.1 (±13.6)	-0.3 (-1.3, 0.8)
GG	88.2 (±18.0)	0.1 (-6.4, 6.5)	100.7 (±13.3)	0.4 (-1.2, 2.0)

Data are collected from HUNT2 (inclusion) and HUNT3 (end of follow-up) for control groups 1 and 2. Data are presented as mean±SD. No missing values unless otherwise stated. HUNT: The Trøndelag Health Study. FVC: Forced vital capacity. *: Control group with chronic obstructive pulmonary disease. †: Control group without lung disease.

Table S2. Sarcoidosis patients and 50 healthy controls from Essen, Germany

**Variable**	**Sarcoidosis**	**Healthy controls**
**Number of cases**, n	112	50
**Age,** y	42±12	51±16
**Gender** (male/female), n (%)	67 (60) / 45 (40)	28 (56) / 22 (44)
**BMI**, kg/m², median (IQR)	28.0 (24.6 – 31.5)	n.a.
**Smoking history** (never/ex/current), n (%)	16 (14) / 25 (22) / 7 (6)	n.a.
**Pulmonary function test**		
FVC, %pred	88.1±18.3	90±23
DLco, %pred	72.1±14.0	85±13
**Organ involvement**		
Pure lung involvement, n (%)	51 (46)	n.a.
≥ 2 organs including lungs, n (%)	34 (30)	n.a.
No lung involvement, n (%)	27 (24)	n.a.

**Organ involvement other than lung**		
Skin, n (%)	21 (19)	n.a.
Liver, n (%)	6 (5)	n.a.
Heart, n (%)	12 (11)	n.a.
Other, n (%)	25 (22)	n.a.
**Treatment,** yes/no (%)	97/15 (87/13)	n.a.
Steroid alone n (%)	54 (48)	n.a.
Steroid in combination n (%)	43 (39)	n.a.
No treatment, n (%)	15 (13)	n.a.
ACE activity (U/L), median (IQR)	43 ± 40	28±13

Crude annual change in lung function by genotype

In the German nLS G+ patients, the annual change in FVC was -0.00 (±3.3) and DLco -0.5 (±4.8) % pred. In homozygous AA, annual FVC and DLco declined with -1.9 (±3.1) and -0.8 (±2.7), respectively.

In the Norwegian nLS G+ patients, the annual change in FVC was 0.2 (±8.7) and DLco 0.4 (±5.9) % pred. In homozygous AA, the annual change was similar. For FVC, the annual change was -0.2 (±4.5) and for DLco the annual change was 0.8 (±4.9).

Figure S1. Annual decline in FVC and DLco according to genotypes AA/G+ in German and Norwegian nLS patients

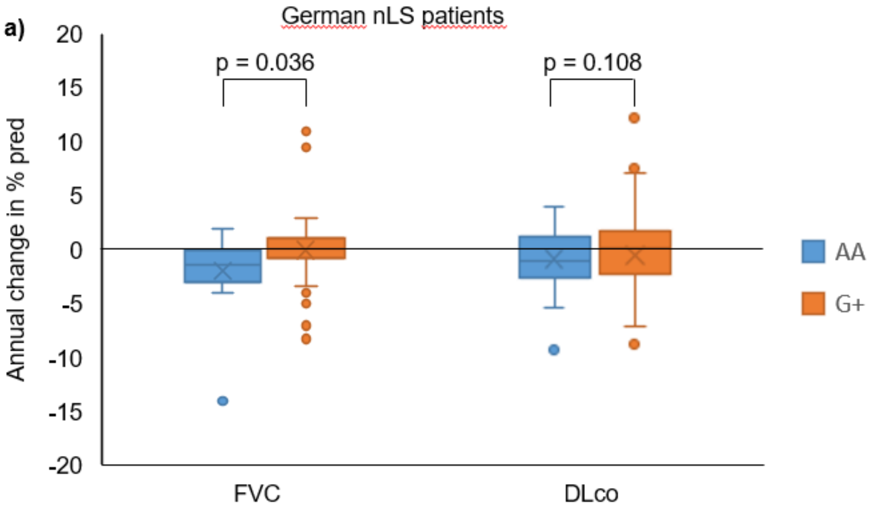

Mean annual change in FVC and DLco in % pred according to homozygous A and G allele (G+) in the German nLS patients. The X in the box represents the mean, the line represents the median.

b)

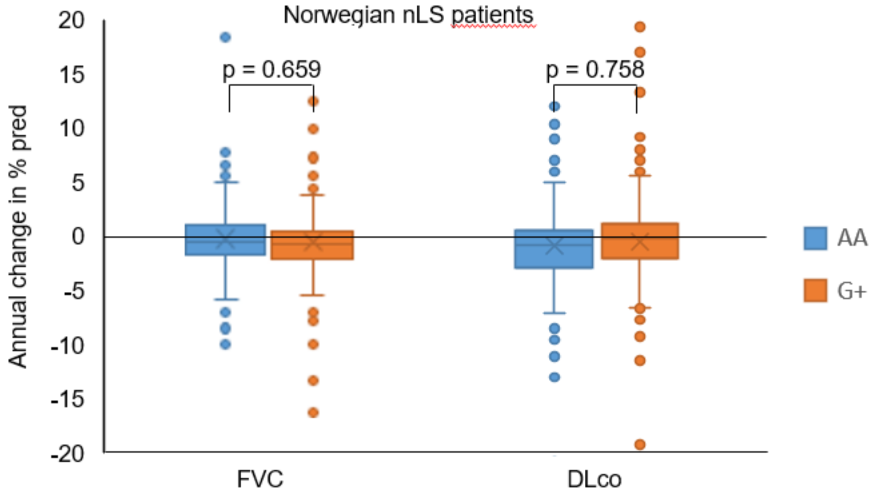

Mean annual change in FVC and DLco in % pred according to homozygous A and G allele (G+) in the Norwegian nLS patients. The X in the box represents the mean, the line represents the median.

Absolute and annual change in FVC in all four study populations

Figure S2 Mean absolute change in lung function (FVC) in all four study populations

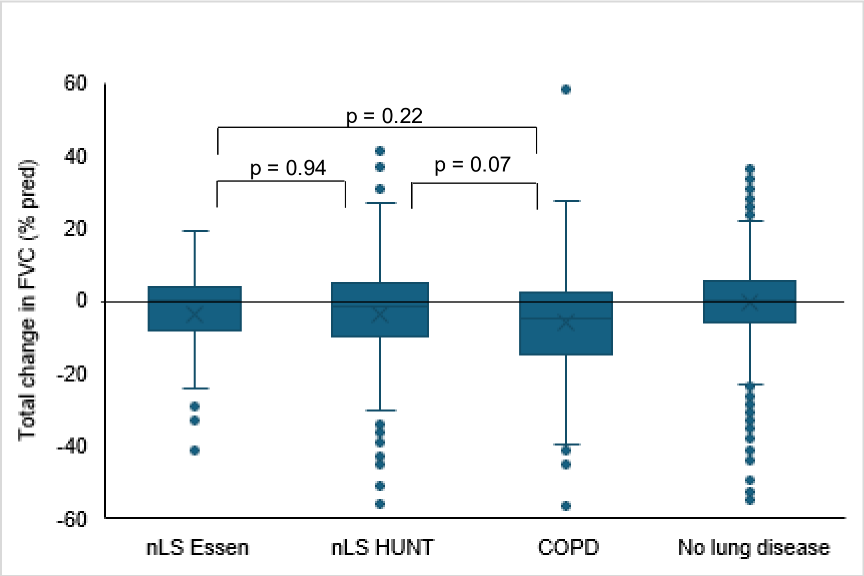

Change in FVC (%pred) from inclusion to end of follow-up (6.6 years for nLS Essen, 8.1 years for nLS HUNT and 10 years for COPD and people with no lung disease. The X in the box represents the mean, the line represents the median.

Figure S3. Mean annual change in lung function (FVC) in all four study populations

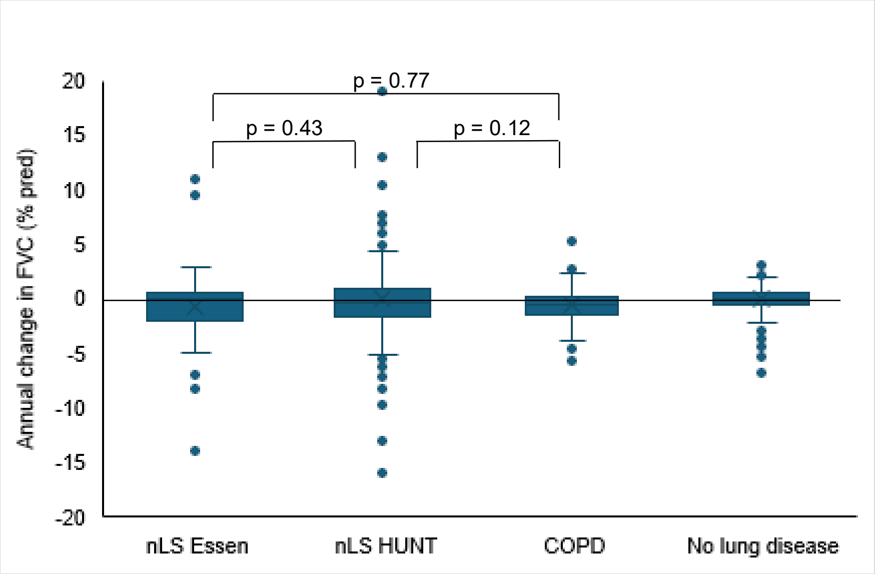

Mean annual change in FVC (%pred) from inclusion to end of follow-up (6.6 years for nLS Essen, 8.1 years for nLS HUNT and 10 years for COPD and people with no lung disease. The X in the box represents the mean, the line represents the median.
